# The immunopathological crosstalk of diabetic periodontitis: Single-cell insights into monocyte dysregulation

**DOI:** 10.1371/journal.pone.0341333

**Published:** 2026-02-17

**Authors:** Chenli Si, Xinlei Wu, Huijuan Cheng, Wentao Jiang, Huijiao Yan

**Affiliations:** 1 Department of Stomatology, Gongli Hospital of Shanghai Pudong New Area, Shanghai, China; 2 Department of Dermatology, Shanghai Ninth People’s Hospital, Shanghai Jiao Tong University School of Medicine, Shanghai, China; 3 School of the 2nd Clinical Medical Sciences, Wenzhou Medical University, Wenzhou, Zhejiang, China; 4 Department of Prosthodontics, Shanghai Ninth People’s Hospital, Shanghai Jiao Tong University School of Medicine, College of Stomatology, Shanghai Jiao Tong University, Shanghai, China; 5 Department of Conservative Dentistry and Endodontics, Shanghai Stomatological Hospital & School of Stomatology, Fudan University, Shanghai, China; Nova Southeastern University, UNITED STATES OF AMERICA

## Abstract

Periodontitis (PD) and type 2 diabetes mellitus (DM) are bidirectionally associated through shared chronic inflammatory mechanisms. Although monocytes serve as central immune mediators in both pathologies, their functional heterogeneity and dynamic alterations in diabetic periodontitis (PDDM) remain poorly characterized. Utilizing single-cell RNA sequencing (scRNA-seq) of peripheral blood mononuclear cells (PBMCs) from healthy controls, PD patients, and PDDM subjects, we systematically investigated the immunopathological crosstalk between these comorbidities. Our analysis revealed a significant shift in monocyte subpopulations, with PDDM patients exhibiting increased classical monocytes (CD14++CD16−) and decreased nonclassical monocytes (CD14 + CD16++) compared to PD counterparts. Functional profiling demonstrated PDDM-enriched classical monocytes upregulated oxygen transport pathways while suppressing chemotaxis and cytokine responses, whereas nonclassical monocytes showed impaired oxidative phosphorylation and nucleotide biosynthesis. Cell communication analysis identified reduced activity of TGF-β signaling and enhanced CCL pathway activation, collectively promoting chronic inflammation and tissue destruction. Regulatory network reconstruction revealed transcription factors (PBX1, TAL1, IRF9) governing monocyte differentiation defects and hyperinflammatory phenotypes. These results suggest mechanistic links how diabetic conditions exacerbate periodontal inflammation through monocyte reprogramming and signaling pathway dysregulation, providing a cellular roadmap for developing targeted immunotherapies in PDDM management.

## 1. Introduction

Emerging evidence substantiates the critical interdependence between oral pathologies and systemic health conditions, with oral diseases affecting over 3.5 billion individuals globally and serving as modifiable risk factors for cardiovascular disorders, metabolic dysregulation, and adverse pregnancy outcomes [1]. This paradigm shift recognizes the oral cavity as a microbial-endocrine-immune interface where dysbiotic microbiota initiate local destruction while disseminating inflammatory mediators systemically. Among oral diseases, periodontitis (PD) – a chronic inflammatory condition induced by dysbiotic microbial communities – exemplifies this systemic connection through its established associations with type 2 diabetes mellitus (T2DM) [2]. Characterized by progressive destruction of tooth-supporting structures, PD affects 19% of adults worldwide and functions as a persistent source of low-grade inflammation [3]. The disease’s systemic impact stems from hematogenous dissemination of periodontal pathogens and virulence factors, triggering elevated circulating inflammatory cytokines that potentiate metabolic dysfunction. Similarly, T2DM - affecting 10.5% of the global population – establishes a proinflammatory milieu through chronic hyperglycemia-induced oxidative stress, marked by sustained elevation of IL-6, TNF-α, and monocyte chemoattractant proteins. This metabolic-inflammatory axis induces microvascular complications and impairs tissue repair capacity, particularly in periodontal compartments [4]. This bidirectional pathophysiology involves dysregulated innate immune responses, where monocytes emerge as pivotal cellular mediators coordinating both metabolic and periodontal inflammation. However, the precise mechanisms underlying monocyte functional reprogramming at the intersection of PD and T2DM remain underexplored, representing a critical knowledge gap in understanding their synergistic pathogenesis.

As central orchestrators of innate immunity, monocytes exhibit functional plasticity that critically modulates inflammatory progression and resolution in both PD and DM [5]. Central to this interplay are monocytes, innate immune cells that orchestrate inflammatory responses and tissue homeostasis. Monocytes can be divided into classical monocytes that highly express CD14 and do not express CD16 (CD14++CD16-), and non-classical monocytes with low – level CD14 and high – level CD16 (CD14 + CD16++) [6]. Classical monocytes are abundant in the bloodstream and are proficient in phagocytosis, playing a crucial role in the early stage of the innate immune response by recognizing and eliminating pathogens. They also participate in antigen presentation through the expression of molecules like HLA-DQB1, which can trigger immunological responses. Non-classical monocytes, although present in relatively lower numbers, are involved in inflammation and tissue repair processes. In chronic inflammatory states such as PD and DM, imbalances in these subsets have been implicated in disease progression [7]. Research has found that patients with chronic periodontitis have more CD14 + CD16++ monocytes and macrophages in their blood and gum. This leads to an increased release of substances that cause inflammation [8]. Also, a substance that helps reduce inflammation called JMJD3 was found to be not working well in the blood cells from the gums of type 2 diabetes patients who have gum disease [8,9]. The work has explained the association of periodontitis with monocytes and might provide an insight into the pathophysiology of periodontitis besides acting as a guide for better development of the treatments for the disease in future. One approach has been transcriptomic analysis in order to determine the immunological links between periodontitis (PD) and diabetes mellitus (DM) [10]. However, such immunological features, such as interaction of signaling between immune cells, could not be explored with bulk RNA sequencing (RNA-seq). This study employs single-cell RNA sequencing (scRNA-seq) to analyze the gene expression profiles of classical and nonclassical monocytes in diabetic periodontitis (PDDM). By integrating functional enrichment, cell-cell communication, and regulatory network analyses, we provide a comprehensive framework for understanding the immunopathological synergy between PD and DM from the systemic (PBMC/monocyte). While prior single-cell studies have predominantly focused on gingival tissue-resident macrophages to elucidate local immunopathology in diabetic periodontitis, our approach emphasizes the systemic immune landscape through peripheral blood monocyte profiling.

## 2. Methods

This study is a secondary analysis of publicly available single-cell RNA-seq data (GEO: GSE244515). The original study was conducted in accordance with the Declaration of Helsinki and approved by the Institutional Review Board of the relevant institution, with written informed consent obtained from all participants. No additional ethical approval was required for this re-analysis

### 2.1. Single-cell data processing and integration

Raw gene expression matrices were retrieved from the Gene Expression Omnibus (GEO). 11 healthy control subjects, 10 patients with PD without DM, and 6 patients with PDDM. The scRNA-seq dataset (GEO) comprises peripheral blood mononuclear cells (PBMCs) from 27 donors, from three distinct groups 11 healthy control subjects, 10 patients with PD without DM, and 6 patients with PDDM. Initial quality control, filtering, and normalization were performed using the Seurat package (v4.3.2) in R (v4.2.3). The filtered dataset was normalized using the sctransform method to remove technical artifacts and regress out the influence of mitochondrial read percentage. To mitigate batch effects and account for inter-individual variability, data integration was performed using the Seurat integration workflow. Specifically, we followed the procedure described by Kang [11] which involves selecting integration anchors based on a mutually defined reference dataset. We specified a reference dataset consisting of cells from healthy individuals within the same GSE244515 cohort. This approach effectively aligns cells across different batches and donors, removing technical variations while preserving biologically relevant heterogeneity. Quality control (QC) was performed by filtering out cells based on the following criteria: fewer than 250 or more than 5000 detected genes, over 15% mitochondrial gene expression, or extreme library sizes. Specifically, cells with gene counts below 500 and more than 15000 were excluded to eliminate potential dead cells and doublets. To identify and remove potential doublets from the single-cell RNA-seq data, we employed DoubletFinder v2.0.3. The algorithm was run on each sample individually using the standard workflow. The expected doublet rate was calculated based on the cell recovery count for each library. The optimal pK parameter was determined empirically, and cells predicted as doublets were filtered out prior to downstream integrative analysis.

### 2.2. Data normalization and scaling

After QC filtering, the expression matrices were normalized using Seurat’ s `NormalizeData` function with the default log-normalization method. This process normalizes gene expression measurements for each cell based on the total expression, multiplies by a scale factor (10,000 by default), and applies a log transformation to the result. The data were then scaled using the `ScaleData` function to regress out unwanted sources of variations, including the number of detected molecules per cell and the percentage of mitochondrial gene expression.

### 2.3. Dimensionality reduction and clustering

Highly variable genes were identified using Seurat’s `FindVariableFeatures` function, selecting the top 2,000 genes based on variance. Principal component analysis (PCA) was performed on the scaled data, retaining the first 20 principal components were retained for downstream analyses. To determine the optimal number of principal components was determined by examining an elbow plot. The data were then integrated into a shared nearest neighbor (SNN) graph using the `FindNeighbors` function, followed by graph-based unsupervised clustering with the `FindClusters` function. Uniform Manifold Approximation and Projection (UMAP) was then utilized visualize the clustered cells in two-dimensional space [12].

### 2.4. Differential expression analysis

Differential gene expression between clusters was analyzed using Seurat’s `FindMarkers` function, which identifies genes that are significantly upregulated in one cluster compared to others. Criteria for differential expression included a log-fold change threshold of 1 and an adjusted p-value cutoff of 0.05 to ensure statistical significance and biological relevance.

### 2.5. Functional enrichment analysis

To elucidate the biological functions and pathways associated with differentially expressed genes, Gene Ontology (GO) and Kyoto Encyclopedia of Genes and Genomes (KEGG) pathway analyses were performed using the clusterProfiler package. The `enrichGO` and `enrichKEGG` functions were applied, and the results were visualized using dot plots and bar plots to highlight significant biological processes and pathways.

### 2.6. Weighted gene co-expression network analysis (scWGCNA)

The scWGCNA package was employed to construct gene co-expression networks from the single-cell data. The `blockwiseModules` function indentified modules of highly correlated genes were identified using the `blockwiseModules` function, enabling the detection of gene clusters associated with specific cell states or phenotypes. Module-trait relationships were explored to link gene modules with biological variables of interest.

### 2.7. Trajectory inference with monocle

Monocle (v2) was utilized for pseudotime trajectory analysis to reconstruct cellular differentiation pathways. Cells were ordered along a trajectory based on their gene expression profiles, facilitating the identification of key genes involved in cell fate decisions and temporal gene expression changes during differentiation.

### 2.8. Cell-Cell communication analysis with CellChat

The CellChat package (v1.1) was used to infer and analyze cell-cell communication networks. Ligand-receptor interactions were identified by comparing the expression of known ligand and receptor pairs across different cell types. Using the `identifyCommunication` and `computeCommunProb` functions were applied to construct communication networks were constructed, and pathway analysis was performed to elucidate signaling pathways mediating intercellular interactions.

### 2.9. Regulatory network analysis with SCENIC

SCENIC (v1.1) was implemented to infer gene regulatory networks and identify active transcription factors (TFs) within each cell cluster. The workflow began with the identification of co-expressed genes using the `GENIE3` or `GRNBoost` algorithms, followed by the determination of regulons through the `RcisTarget` module. Lastly, the activity of each regulon was scored per cell using the `AUCell` method, characterizing the regulatory programs that drive cellular heterogeneity.

## 3. Results

### 3.1. scRNA-seq map landscape of PBMCs

**Fig 1:**
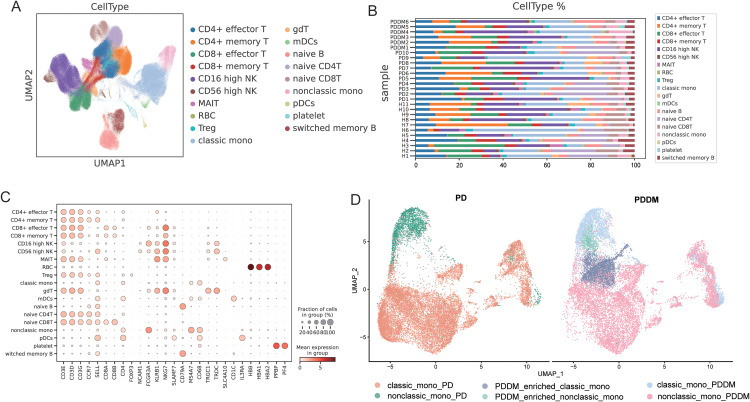
Peripheral blood mononuclear cells (PBMCs) of healthy donors (H) and of patients with periodontitis (PD) and both PD and diabetes mellitus (DM) (PDDM). **(A, B)** UMAP plot showing cell clusters distinguished by gene expression signatures. The cell compartments consist of naïve T cells, effector and memory T cells, regulatory T cells (Treg), natural killer (NK) cells, naïve B cells, switched memory B cells, classical monocytes (classic mono), intermediate monocytes (intermediate mono), non-classical monocytes (nonclassic mono), gamma delta T cells (gdT), mucosal-associated invariant T cells (MAIT), myeloid dendritic cells (mDC), plasmacytoid DCs (pDC), red blood cells (RBC) and platelets. **(C)** Bubble diagram showing the top 5 genes across distinct cell types. **(D)** UMAP plot showing monocyte clusters distinguished by unsupervised clustering analysis.

We processed the scRNA-seq data, which included samples from 11 healthy donors, 10 PD patients, and 6 PDDM patients, retaining a total of 243,038 cells after quality control filtering. Following principal component analysis (PCA), unsupervised clustering combined with two-dimensional uniform manifold approximation and projection (UMAP) revealed 19 molecularly distinct clusters, identified based on gene expression patterns relative to other cells. The resulting cell clusters were annotated according to cell types using canonical cell markers, as described previously [11] (Fig 1A). This analysis identified distinct clusters, including CD4 + effector T, CD4 + memory T, CD8 + effector T, CD8 + memory T, CD16 high NK, CD56 high NK MAIT, RBC, Treg, classical monocytes, gdT, mDCs, naive B, naive CD4T, naive CD8T, nonclassical monocytes, pDCs, platelets, and switched memory B cells. Compared to PD, the proportion of classical monocytes increased in PDDM, while nonclassical monocytes decreased (Fig 1B). Therefore, we further grouped the monocytes and identified, through unbiased clustering, two PDDM-enriched monocyte groups: PDDM-enriched classical monocytes and PDDM-enriched nonclassical monocytes (Fig 1C).

### 3.2. Enriched pathways and signaling in monocytes in PDDM

**Fig 2:**
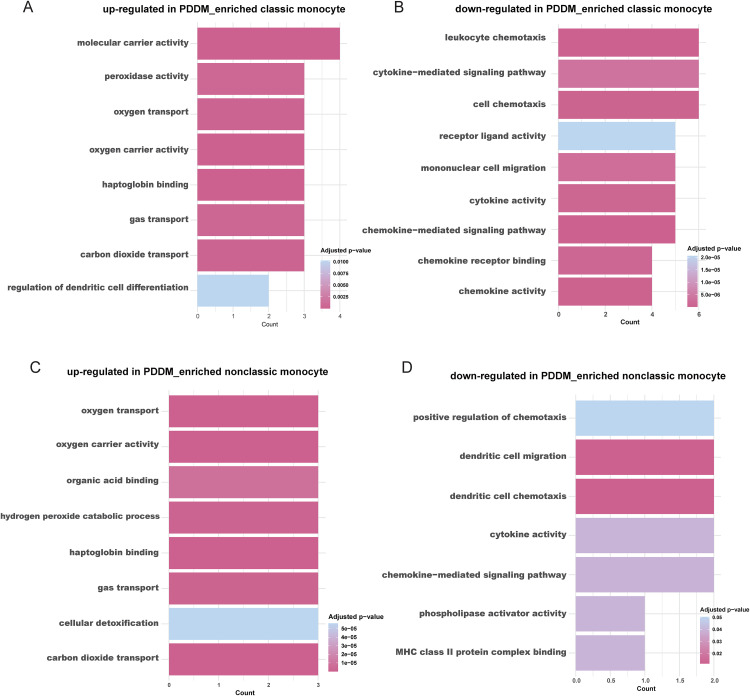
GO enrichment analyses of DEGs. **(A)** Bar plot of GO analysis in up-regulated DEGs in PDDM_enriched classic monocyte. **(B)** Bar plot of GO analysis in down-regulated DEGs in PDDM_enriched classic monocyte. **(C)** Bar plot of GO analysis in up-regulated DEGs in PDDM_enriched nonclassic monocyte. **(D)** Bar plot of GO analysis in down-regulated DEGs in PDDM_enriched nonclassic monocyte. DEGs, differentially expressed genes; GO, Gene Ontology.

biological processes related to hemoglobin function, including carbon dioxide and oxygen transport, were significantly upregulated (Fig 2A). Conversely, processes related to cellular chemotaxis and cytokine-mediated processes were downregulated (Fig 2B). A similar pattern was observed PDDM- enriched nonclassical monocytes, where hemoglobin-related processes were also elevated (Fig 2C). However, in these nonclassical monocytes, processes such as ribonucleotide and ribose phosphate biosynthesis, as well as oxidative phosphorylation enzyme activity, showed a decreasing trend (Fig 2D). Overall, the functional characteristics of PDDM-enriched monocytes in both groups differed from those of DM monocytes, with an increase in hemoglobin-related functions in both groups.

### 3.3. Identification of key module genes in monocytes in PDDM

**Fig 3:**
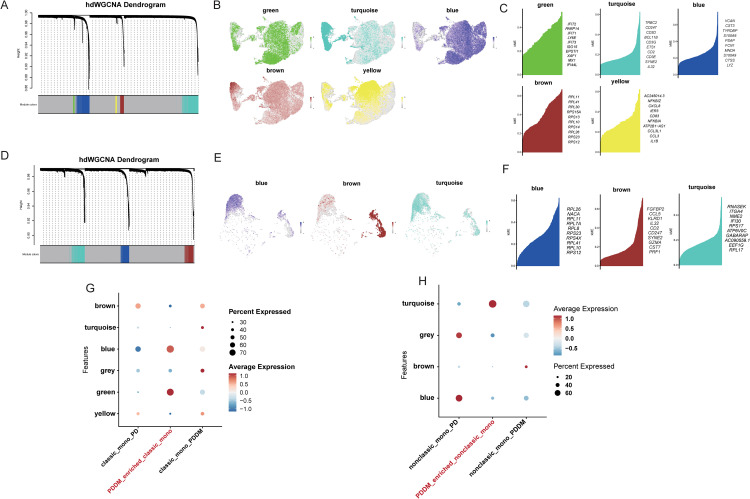
scWGCNA identifies key module genes in classic monocytes and nonclassic monocytes of PD and PDDM patients. **(A)** The matrix plot visually represents the inter-module relationships by depicting the correlation between module genes in classic monocytes. **(B)** UMAP plot of classic monocytes. **(C)** Top feature genes in different modules in classic monocytes. **(D)** The matrix plot visually represents the inter-module relationships by depicting the correlation between module genes in nonclassic monocytes. **(E)** UMAP plot of nonclassic monocytes. **F** Top feature genes in different modules in nonclassic monocytes. **(G, H)** Correlation among feature genes in the module of classic monocytes **(G)** and nonclassic monocytes **(H)**.

We employed scWGCNA to identify module genes specifically expressed in classical and nonclassical monocytes, and to explore their functions. For PDDM-enriched classical monocytes, a soft power value of 8 was used to construct the co-expression network. Based on the scale-free network structure, the final analysis identified five modules, excluding the grey module (Fig 3A). These modules exhibited varying co-expression patterns across different cell clusters (Fig 3B and 3C). We then assessed the module scores for the five clusters and found that the turquoise and green modules were highly activated in PDDM-enriched classic monocytes (Fig 3G). The same analysis was conducted in nonclassic monocytes, where the turquoise module was highly activated in PDDM-enriched nonclassic monocytes (Fig 3D, 3E, 3H).

**Fig 4:**
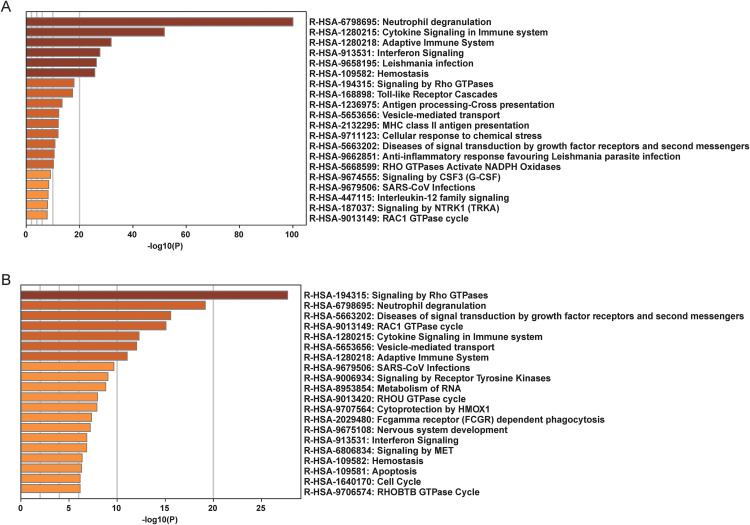
Enrichment analyses of key module genes in classic monocytes and nonclassic monocytes. **(A)** Barplot of enrichment of genes in turquoise and green modules in classic monocytes, colored by p-value. **(B)** Barplot of enrichment of genes in turquoise module in nonclassic monocytes, colored by p-value.

Based on these results, we performed enrichment analysis for the genes within the highly activated modules. Notably, REACTOME enrichment analysis indicated that these genes were enriched in pathways related to Interferon Signaling, growth factor receptor signaling, and processes that highlight the intricate balance between cell proliferation, survival, and response to external threats (Fig 4A and 4B).

### 3.4. Identification of interactive pathways in PDDM and PD

**Fig 5:**
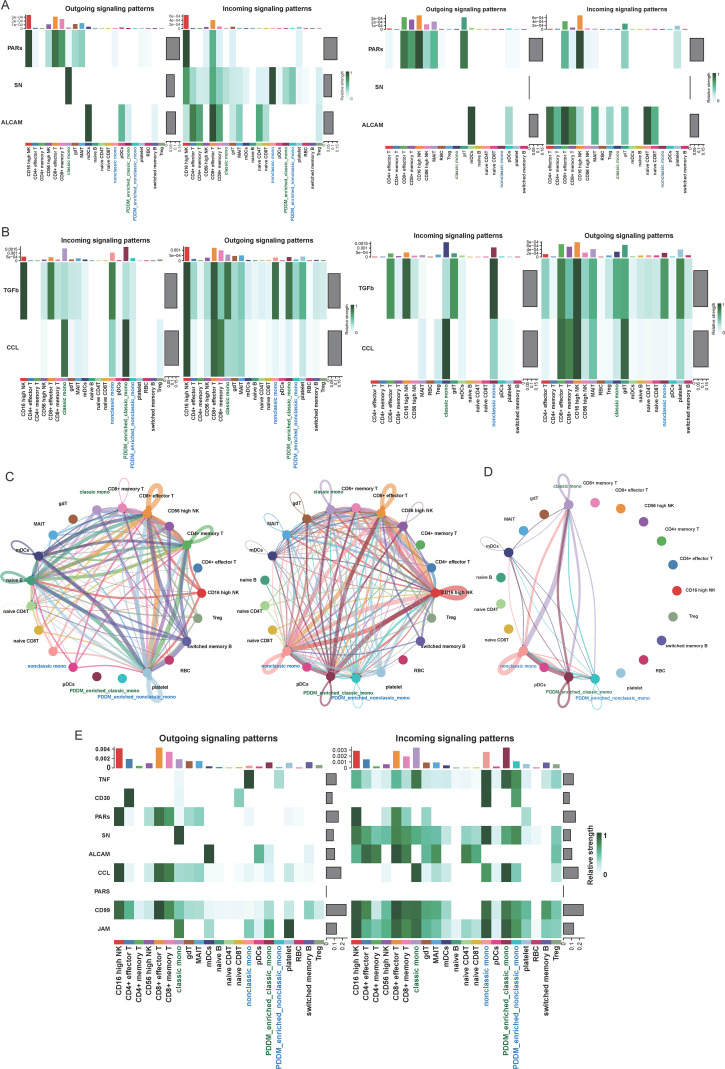
Single-cell transcriptional analysis reveals the cell–cell crosstalk network. **(A)** The outgoing signaling and incoming signaling of PARs, SN, and ALCAM signaling pathways in PD and PDDM. **(B)** The outgoing signaling and incoming signaling of TGF-β and CCL signaling pathways in PD and PDDM. **(C)** Analysis of the number of interactions and interaction strength among different cell types in PDDM. **(D)** Circle plot of TGF-β signalling in PDDM. The strength of the crosstalk is correlated with the edge width. **(E)** Overview of the outgoing signaling and incoming signaling in PDDM.

CellChat was employed to discern the differential overexpression of ligands and receptors across various cell cohorts. The results showed that both the number and potency of interactions were significantly enhanced in PDDM patients compared to those with PD, especially in the interactions between monocytes (including both classic and nonclassic monocytes) and other immune cells. However, interactions between DCs and other immune cells were relatively reduced (S1 File). This finding helps elucidate the potential influence of monocytes on PDDM onset. The subsequent analysis with CellChat focused on relevant molecules, receptors, and pathways. Cell–cell communication analysis revealed differentially enriched pathways, including PARs, SN, and ALCAM, in monocyte subtypes that were significantly enriched only in PDDM compared to PD (Fig 5A). Moreover, a significant difference was observed in the relative strength between the TGF-β and CCL signaling pathways in PD and PDDM (Fig 5B). Previous studies have shown that TGF-β plays a crucial role in the pathogenesis of PD and PDDM by modulating inflammation, immune responses, and tissue regeneration. Further investigation revealed that PDDM had lower levels of TGF-β among monocyte subpopulations, indicating the presence of chronic inflammation. Additionally, signaling associations were detected in PDDM-enriched classic monocytes and pDCs, which had not been observed in classic monocytes and pDCs. A similar trend was observed in PDDM-enriched nonclassic monocytes and mDCs (Fig 5C and 5D). Fig 5E illustrates the signaling pathways where the expression profiles of PDDM-enriched classic and nonclassic monocytes differ from those of classic and nonclassic monocytes. The reduction of these signaling pathways indicates a dampened immune response in PDDM-enriched classic monocytes, while the decreases observed in PDDM-enriched nonclassic monocytes suggest weaker chemotaxis and tissue repair capacity. In summary, the alterations in chemotaxis and inflammatory pathways in PDDM-enriched monocyte subtypes likely exacerbate PDDM-related damage.

### 3.5. Analysis of transcriptional regulators of monocytes and DCs in PDDM and PD

**Fig 6:**
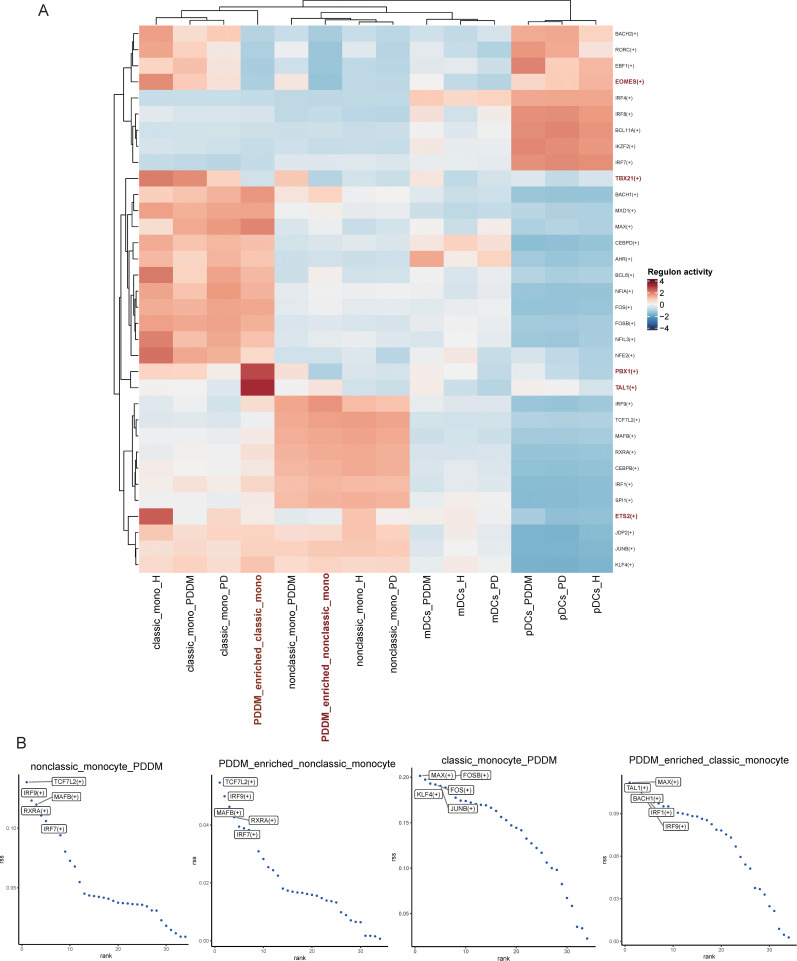
Analysis of key regulators in monocytes subtypes. **(A)** Heatmap plot displaying the representative regulatory elements in each cell types. **(B)** This plot shows the RSSs of various regulatory elements across different monocyte subsets, with the data points representing different regulatory elements.

To identify key regulators of these cell types, we assessed the activity of each regulon across all monocyte and DC subtypes. The five regulators involved in maintaining cell identity are shown in Fig 6. For PDDM-enriched classic monocytes, the five TFs were MAX, TAL1, BACH1, IRF1, and IRF9, while for PDDM-enriched nonclassic monocytes, they were TCF7L2, IRF9, MAFB, RXRA, and IRF7. Fig 6A displays a heatmap highlighting two transcription factors, PBX1 and TAL1, which are highly expressed in PDDM-enriched classic monocytes.

## 4. Discussion

Gram-negative obligate anaerobes cause PD, an infectious and inflammatory periodontal disease. Epidemiological studies have shown demonstrated that individuals with DM have approximately a threefold increased risk of developing PD compared to those without DM [13,14]. The severity of periodontitis worsens in a high-glucose environment, leading to dysregulation of the host immune-inflammatory response. Moreover, lipopolysaccharides present in the cell walls of Gram-negative bacteria trigger inflammatory mediators under hyperglycemic conditions [15–17]. Additionally, PD also increases the risk of developing DM and complicates blood glucose management in DM patients [18]. Consequently, we systematically investigated how single cells regulate peripheral blood in these two patient groups.

Single-strand conformational gene sequencing was performed to examine periodontitis-affected peripheral blood. Our findings reveal that periodontitis alters gingival tissue immunology by altering monocyte inflammatory signaling. The peripheral blood of periodontitis patients and healthy controls had multiple monocyte groups with different activation states. Using unbiased clustering, we observed two categories of monocytes in people with PDDM: classical monocytes (CD14++CD16-) and non-classical monocytes (CD14 + CD16++). We utilized “FindMarkers” to identify marker genes in these two cell types for GO and KEGG pathway enrichment analysis. PDDM-enriched classic monocytes had high HLA-DQB1 and IFI30 levels. HLA-DQB1, an MHC class II member, improves antigen presentation, which triggers immunological responses [19]. Upregulation of IFI30, which takes part in the linked lysosomal processing of antigens, speaks to increased antigen presentation capabilities [20]. The classical monocyte population exhibited significantly elevated expression levels of HLA-DQB1, suggesting an enhanced antigen-presenting capacity for antigen presentation. This upregulation likely reflects improved immune surveillance capabilities and more robust immune responses relative to other monocyte subsets [21,22]. Additionally, we found that both PDDM-enriched classic and nonclassic monocytes had lower levels of HLA-DRB5 expression. This result is consistent with study by Restrepo et al., which found that type II DM often results in a reduction in HLA-DR expression [23]. The elevated expression of HLA-DQB1 and IFI30 in classical monocytes may reflect a compensatory regulatory mechanism aimed at preserving immune homeostasis and maintaining immunological competence in PDDM. In addition, in PDDM-enriched classical monocytes, the expressions of TMEM176B and TMEM176A regulating dendritic cell (DC) function and antigen presentation are up-regulated [24]. The results suggest that this cell type may has improved immune regulation and antigen presentation.

We found that EGR1 and RETN were highly expressed in PDDM-enriched nonclassical monocytes. Collagenase expression in periodontal tissue and wound healing capacity are influenced by EGR1, which is linked to inflammation and tissue remodeling [25,26]. Hivert et al. found that RETN is a pro-inflammatory molecule that increases cytokine release and exacerbates inflammation via promoting insulin resistance in DM [27]. Collectively, these findings suggest that non-classical monocytes, depleted in PDDM, as potential key mediators of chronic inflammatory pathogenesis. Notably, our analysis identified impaired chemotactic activity in both classical and non-classical monocyte subsets, potentially contributing to disrupted tissue repair processes and the persistence of chronic inflammation within periodontal tissues. In addition, HBB, HBA2, and HBA1 were also expressed more in both populations. Gene products of these genes are important components of hemoglobin and when expressed in nonerythroid cells, protect against oxidative stress [28]. These gene expression profiles may be related to disease progression and immune function in PDDM.

Next, we identified unique signaling pathways involved in different cell interactions. SN plays a crucial role in modulating immune responses and pathogen interactions, as its ligand SIGLEC1 acts as a phagocytic receptor on macrophages, binding to sialylated bacteria to promote phagocytosis and antigen presentation [29,30]. Our investigation suggested distinct patterns of signaling pathway activation patterns between monocyte subsets in patients with PDDM. Specifically, classical monocytes exhibited significant activation of SN signaling pathways, a feature uniquely observed in PDDM and absent in PD alone. Additionally, innon-classical monocytes enriched in PDDM, we detected functional expression of PAR signaling receptors, which critically regulate innate immune responses in gingival epithelial cells to maintain immunological homeostasis. Additionally, ALCAM, a member of the immunoglobulin superfamily known for its role in cell adhesion and migration, was found exclusively in PDDM-enriched classical monocytes, indicating distinct immunomodulatory functions between these two cell types. Subsequent analysis identified significant differences in two signaling pathways with significant differences between PDDM-enriched monocytes and monocytes in PDDM patients. As TGF-β, a negative regulator that maintains immune homeostasis, can lead to hyperactive immune responses when its signaling is deficient [31]. In TGF-β1-null mice, deficient TGF-β signaling is associated with elevated levels of inflammatory factors, such as CCL3 and TNF-α [32]. Consistent with this, our data pointed to a high level of CCL signaling but a low level of TGF-β signaling in both PDDM patients and PDDM-enriched monocytes, suggesting the persistence of chronic inflammatory states in PDDM and demonstrating a pro-inflammatory function. This pathway has provided specific communications between the PDDM-enriched monocytes and DCs, which may promote the migration of DCs toward the sites of inflammation, affect antigen presentation, and maybe enhance the production of inflammatory factors involved in much stronger immune responses in PDDM.

Finding key transcription factors (TFs) like TAL1, TCF7L2, IRF9, and PBX1 implied the intricate regulatory systems that control various cell types. A different transcriptional landscape in PDDM-enriched classical monocytes is suggested by the differential expression of these TFs, with PBX1 and TAL1 being specifically upregulated in these cells. A homeodomain transcription factor involved in the formation of myeloid cells is called PBX1 [33], which is known to restrain myeloid maturation in hematopoietic progenitors [34]. Consequently, the observed overexpression patterns in classical monocytes enriched in PDDM may be mechanistically associated with their incomplete differentiation state and enhanced antigen-presenting capabilities, potentially reflecting a compensatory adaptation during disease progression. Notably, the myeloid developmental factor TAL1 has been previously shown to regulate the cell cycle in monocytic precursors and influence the differentiation of myeloid progenitor cells [35,36]. By elucidating the monocyte-specific transcriptional and functional reprogramming that occurs under diabetic conditions, this work provides a cellular and molecular basis for developing targeted immunomodulatory therapies. The identification of dysregulated monocyte subpopulations—particularly the increase in classical monocytes with enhanced antigen-presenting capacity and the decrease in nonclassical monocytes with impaired tissue repair functions—offers potential biomarkers for early diagnosis and stratification of PDDM patients. Moreover, the disrupted TGF-β and CCL signaling pathways highlight actionable targets for restoring immune homeostasis. Transcription factors such as PBX1 and TAL1, which drive monocyte dysregulation, may serve as novel therapeutic targets to modulate myeloid differentiation and function.

Our study investigates monocyte dysregulation in diabetic periodontitis (PDDM) via peripheral blood mononuclear cell (PBMC) single-cell RNA sequencing (scRNA-seq), while Agrafioti et al. [37] focuses on gingival tissue-resident macrophages, with both studies confirming T2DM exacerbates periodontal inflammation—we found reduced TGF-β and enhanced CCL signaling in monocytes, and they observed RELA (NF-κB subunit) hyperactivation in macrophages, plus neither supports binary immune cell polarization models. Key differences emerge from cellular and tissue specificity: we identified PDDM-specific monocyte subset shifts (increased CD14++CD16 − , decreased CD14 + CD16++) and regulators like PBX1/TAL1, while they defined 5 macrophage subsets (e.g., MHCIIhighCD44+), highlighted CIITA/RUNX2 as core transcription factors, and uncovered macrophage-T cell crosstalk via PVR-TIGIT. These discrepancies reflect systemic (monocytes) vs. local (macrophages) immune responses, together emphasizing the need to target both compartments for PDDM therapy.

The main limitations of this study lie in its relatively small sample size (n = 27) and the lack of functional validation. First, it concentrated on the transcriptome of PBMCs, excluding the transcriptome information from neutrophils, which are the first immune cells recruited to periodontal sites. Therefore, the differences in neutrophils in PDDM/PD need further exploration. Second, the study included 11 healthy donors, 10 PD patients, and 6 PDDM patients, with a relatively small sample size and limited representation of demographic characteristics (such as age, gender, ethnic background, and comorbidities other than DM and PD) among the subjects. This diversity in subject characteristics may introduce confounding factors that are difficult to control, potentially affecting the generalizability and reliability of the study results. Third, the current analysis mainly infers functional changes in monocytes (such as altered chemotaxis, cytokine response, and antigen-presenting ability) based on transcriptomic data (e.g., differential gene expression and pathway enrichment). Direct functional verification experiments (e.g., in vitro monocyte chemotaxis assays, cytokine secretion detection, and antigen presentation functional tests) are lacking, which may lead to insufficient confirmation of the inferred functional alterations and limit the robustness of the conclusions regarding monocyte functional reprogramming in PDDM.

In conclusion, our study identified two novel monocyte subtypes, PDDM-enriched classical monocytes and nonclassical monocytes in PDDM patient PBMCs and clarified their biological functions. Our findings provide a new immunological explanation for the complex association between PD and PDDM.

## Supporting information

S1 File Table of abbreviations list are within the Supporting Information files. (DOCX)

S1 Fig Single-cell transcriptional analysis reveals the cell-cell crosstalk network. (TIFF)
