# Supplementary material for: The immunopathological crosstalk of diabetic periodontitis: Single-cell insights into monocyte dysregulation
Source: PLoS One. 2026 Feb 17;21(2):e0341333. doi: 10.1371/journal.pone.0341333 (PMC12912578; doi:10.1371/journal.pone.0341333)
Supplement: S1 File — (DOCX) [file pone.0341333.s001.docx]

**Table1**

**Abbreviations List**

| Abbreviations | Full Name |
| --- | --- |
| PD | Periodontitis |
| DM | Diabetes Mellitus |
| T2DM | Type 2 Diabetes Mellitus |
| PDDM | Diabetic Periodontitis |
| PBMC | Peripheral Blood Mononuclear Cells |
| RNA-seq | RNA Sequencing |
| scRNA-seq | Single-Cell RNA Sequencing |
| TGF-β | Transforming Growth Factor-beta |
| CCL | Chemokine (C-C motif) Ligand |
| PBX1 | Pre-B-cell Leukemia Transcription Factor 1 |
| TAL1 | T-cell Acute Lymphoblastic Leukemia Protein 1 |
| IRF9 | Interferon Regulatory Factor 9 |
| IL-6 | Interleukin-6 |
| TNF-α | Tumor Necrosis Factor-alpha |
| HLA-DQB1 | Human Leukocyte Antigen DQ Beta 1 |
| JMJD3 | Jumonji Domain-Containing Protein 3 |
| QC | Quality Control |
| PCA | Principal Component Analysis |
| SNN | Shared Nearest Neighbor |
| UMAP | Uniform Manifold Approximation and Projection |

**Supporting figure1**


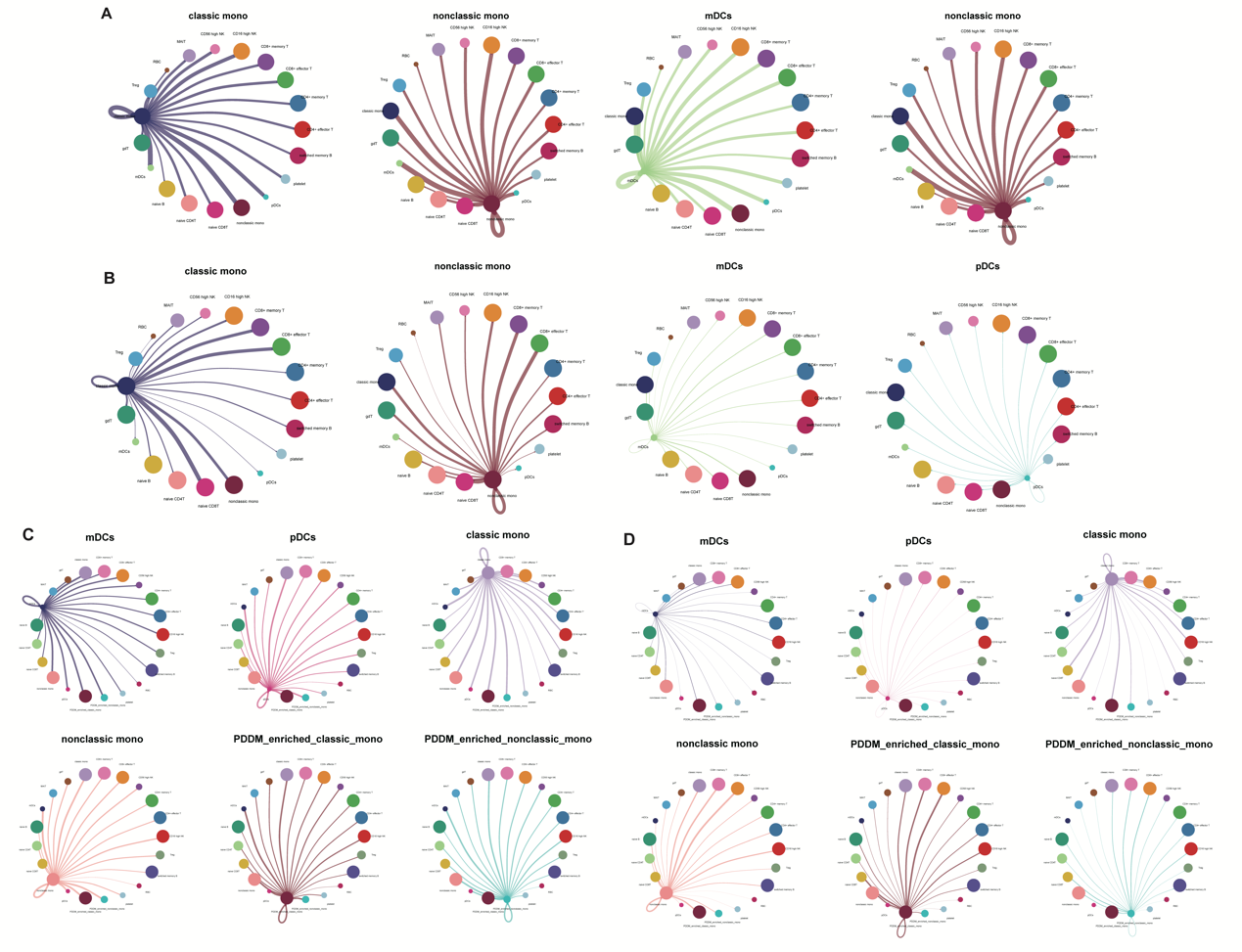


**S1. Single-cell transcriptional analysis reveals the cell–cell crosstalk network**. **A, B** CellChat analysis compared the counts(**A**) and strength(**B**) of interactions between each cluster in PD. **C, D** CellChat analysis compared the counts(**C**) and strength(**D**) of interactions between each cluster in PDDM.
